# Editorial: Deconstructing the Influence of Genetic and Age Vulnerability to Psychiatric Disorders

**DOI:** 10.3389/fpsyt.2019.00013

**Published:** 2019-01-25

**Authors:** Cristina Cadoni, Maria Antonietta De Luca

**Affiliations:** ^1^CNR Institute of Neuroscience, Cagliari Section, National Research Council of Italy, Cagliari, Italy; ^2^Department of Biomedical Sciences, University of Cagliari, Cagliari, Italy

**Keywords:** adolescence, genetic influence, environmental influence, epigenetics, THC - tetrahydrocannabinol, alcohol, substance use disorders (SUD), psychopathologies

The long-standing issue whether human behavior is more influenced by genes or by environment (*nature* vs*. nurture*) has characterized the scientific and social debates in particular in the twentieth century. Decades of genetic studies have highlighted the important contribution of genes in shaping “normal” and pathological phenotypes, but also have evidenced how genes interact with environment thus rendering the study of the genetic and environmental contribution more complex (1, 2). Nowadays we know that environment impacts on genes through epigenetic mechanisms, affecting gene regulation and expression without alteration in DNA sequence (3, 4). Therefore, development of psychopathologies is the result of a complex dynamic interplay between genetic and environmental factors where epigenetic alteration are the biological responses to environmental input and these epigenetic alterations can be transmitted to next generations as in the case of early trauma.

In this already complex picture another factor influencing behavioral phenotype, and thus the emergence of psychopathologies, is the age of the individual. Indeed, the entire developmental period, prenatal and early postnatal, is critical for gene x environment interaction, and events occurring during these periods might have long-lasting and powerful impacts also on next generations as it was recognized long time ago (5, 6). In this regard adolescence, the transition phase between childhood and adulthood, represents a particular critical one (7). In this developmental period an extensive neuroanatomical and functional reorganization occurs in the brain leading to substantial maturational changes in cognition and affect regulation. Thus adolescence is characterized by a heightened emotional reactivity, sensation and novelty seeking, risk taking behavior, sensitivity to peer influence, and impulsivity accompanied by a poor capacity to self-control on actions and emotions. Genetic influences on behavioral phenotypes also change (increase) during adolescence, highlighting the dynamic nature of genes impact on human behavior (8).

Adolescence is also a peak time for clinical onset of most mental illnesses, as well as substance use disorders (9, 10). This is likely due to a developmental mismatch between early maturing (limbic areas) and late maturing (cortical areas) brain structures responsible for typical adolescent behavior and predisposing to onset of psychopathologies (11, 12). Moreover, adolescence is also a time of high stress reactivity and stress response systems (hypothalamic-pituitary-adrenal axis) still maturing (13). All these factors (genetic, environmental, and epigenetic factors), that contribute to shape the brain, could be the reasons why some, but not all, adolescents are at higher risk to develop psychopathological disorders.

The authors of this research topic contributed to clarify the role of genetic and/or environmental factors in increasing the risk for psychopathologies or substance use disorders. Cimino et al. investigates how DNA methylation at the dopamine transporter (DAT) promoter influences risk for psychopathology and the intergenerational transmission between parents and school-age youths. The study explores the correlation between DAT methylation and the presence of psychopathological symptoms in parents and maladaptive emotional-behavioral functioning in children. These results add further evidence about the contribution of genetic and environmental factors in predicting psychopathological symptoms within non-clinical populations.

The debate about the involvement of brain-derived neurotrophic factor (BDNF) in the pathophysiology of major depressive disorder (MDD) has been further analyzed by Kishi et al. who, reviewing several meta-analysis, came to the conclusion that Val66Met polymorphism in the BDNF gene was not associated with MDD but appeared to be related to antidepressant response in patients with MDD. However plasma/serum BDNF levels were lower in patients with acute MDD and antidepressant and electroconvulsive therapy increased plasma/serum BDNF levels. Although Val66Met variation in the BDNF gene does not appear to be a risk factor for MDD, peripheral BDNF levels have been confirmed to be markers for the state of MDD.

One of the most common neurodevelopmental disorders in childhood is attention-deficit hyperactivity disorder (ADHD), characterized by inattention, impulsivity, and hyperactivity (14). The dopaminergic system has been shown to have a role in the etiology of this disorder (15). Although social impairment has not been considered a core feature of this disease, it has been frequently found in ADHD patients (16, 17) having serious implications in everyday life. There is evidence that impairment in social behavior is partly determined by genetic factors. Among these the gene catechol O-methyltransferase (COMT), coding for the enzyme degrading catecholaminergic neurotransmitters, has been implicated, and among the several polymorphisms found for this gene the Val158Met polymorphism has been associated with hyperactivity and inattention symptoms observed in ADHD patients. However, contrasting results appeared in the literature with some supporting a significant effect of the Val allele (18) in children with ADHD while others finding an effect of the Met allele (19). In their article Millenet et al. further analyzed the effects of the COMT gene Val158Met polymorphism on the degree of ADHD symptoms and on social behavior in a large epidemiological population of adolescents with the belief that investigation of gene-phenotype interactions could help in identifying vulnerable phenotypes for ADHD symptomatology.

Alterations in dopamine neurotransmission are not only associated with ADHD but also with obsessive-compulsive disorders (OCD), often associated with ADHD (20). These diseases are characterized by poor decision making and lack of control on executive functions. The underlying neurobiological mechanism of these disorders have been studied through the years by using many animal models. In this Topic Cinque et al. provide a first phenotypic characterization of DAT knockout rats as a promising model for studying ADHD, OCD, and other neuropsychiatric disorders such as compulsive behavioral addictions.

Preclinical research has given an extremely important contribution in disentangling the complex interplay between genes and environment and their contribution to psychopathologies. As regards the risk to develop psychiatric diseases following adolescent cannabis use, several preclinical investigations have provided evidence for a critical role of a reduced prefrontal cortical GABAergic neurotransmission (reviewed by Renard et al.) which seems to be at the basis of behavioral disturbances induced by repeated adolescent use of cannabis products. These results are consistent with a prefrontal GABAergic hypofunction in schizophrenic patients (21–25).

Alcohol Use Disorders (AUD) often originates during adolescence and young adulthood. Clinical studies suggest that early onset of alcohol use is associated with the risk to develop an AUD later in life (26–28). However results from animal studies have been contrasting. Therefore this issue has been further investigated by Labots et al. with the aim of comparing the influence of age of onset of alcohol consumption on control over alcohol seeking in rats.

## Author Contributions

CC wrote the article. MADL critically revised and approved the final version of the manuscript.

### Conflict of Interest Statement

The authors declare that the research was conducted in the absence of any commercial or financial relationships that could be construed as a potential conflict of interest.
